# The Impact of Cold Storage on Adenosine Diphosphate-Mediated Platelet Responsiveness

**DOI:** 10.1055/s-0040-1714254

**Published:** 2020-08-13

**Authors:** Juergen Koessler, Philipp Klingler, Marius Niklaus, Katja Weber, Angela Koessler, Markus Boeck, Anna Kobsar

**Affiliations:** 1 Institute of Clinical Transfusion Medicine and Haemotherapy, University of Wuerzburg, Germany

**Keywords:** platelet physiology, cold storage, adenosine diphosphate, purinergic receptors, inhibitory signaling

## Abstract

**Introduction** Cold storage of platelets is considered to contribute to lower risk of bacterial growth and to more efficient hemostatic capacity. For the optimization of storage strategies, it is required to further elucidate the influence of refrigeration on platelet integrity. This study focused on adenosine diphosphate (ADP)-related platelet responsiveness.

**Materials and Methods** Platelets were prepared from apheresis-derived platelet concentrates or from peripheral whole blood, stored either at room temperature or at 4°C. ADP-induced aggregation was tested with light transmission. Activation markers, purinergic receptor expression, and P2Y12 receptor function were determined by flow cytometry. P2Y1 and P2X1 function was assessed by fluorescence assays, cyclic nucleotide concentrations by immunoassays, and vasodilator-stimulated phosphoprotein (VASP)-phosphorylation levels by Western blot analysis.

**Results** In contrast to room temperature, ADP-induced aggregation was maintained under cold storage for 6 days, associated with elevated activation markers like fibrinogen binding or CD62P expression. Purinergic receptor expression was not essentially different, whereas P2Y1 function deteriorated rapidly at cold storage, but not P2Y12 activity. Inhibitory pathways of cold-stored platelets were characterized by reduced responses to nitric oxide and prostaglandin E1. Refrigeration of citrated whole blood also led to the attenuation of induced inhibition of platelet aggregation, detectable within 24 hours.

**Conclusion** ADP responsiveness is preserved under cold storage for 6 days due to stable P2Y12 activity and concomitant disintegration of inhibitory pathways enabling a higher reactivity of stored platelets. The ideal storage time at cold temperature for the highest hemostatic effect of platelets should be evaluated in further studies.

## Introduction

Platelet transfusions are required for the prevention and therapy of hemorrhage related to thrombocytopenia or platelet disorders. 1 In transfusion medicine, it is an important issue to optimize the ex vivo storage conditions for platelet concentrates (PC) to preserve platelet integrity and to minimize storage lesions. 2 During the last decades, many amendments have been made by adapting container material, storage media, or technical procedures. Depending on country-specific regulations, PC are commonly stored for 4 to 7 days under continuous agitation at room temperature (RT). 3 Until the 1980s, refrigerated PC with storage temperatures of 1 to 6°C have also been considered as components for transfusions. 4 However, the transfusion of cold platelets was associated with reduced platelet survival compared with room temperature platelets. 5 In addition, the number of patients with hypoproliferative thrombocytopenia due to hematological malignancies continuously increased requiring long-circulating platelets. In consequence, room temperature was implemented as the standard for the storage of PC, although bearing a higher risk of bacterial growth. 4 6

Recently, the interest in cold platelets has awakened. Due to their higher responsiveness, they are considered to be advantageous for acute hemorrhage, for example, in cardiovascular surgery 7 8 or in military settings. 4 Studies with cold platelets showed superior aggregation responses to agonists in vitro. 9 10 11 In vivo, cold platelets were able to reduce bleeding more efficiently in individuals under aspirin 12 or in patients with thrombocytopenia. 13 In contrast, other investigations reported that platelet function measured by hypotonic shock response, aggregation, or serotonin uptake is better maintained at RT 14 and that cold storage leads to the loss of discoid shape and to the formation of large pseudopodia. 15

Therefore, for the improvement of storage conditions and for the preparation of clinical studies, it is essential to shed more light upon the biochemical mechanisms associated with cold storage of platelets. Platelet reactivity to adenosine diphosphate (ADP) represents a significant characteristic of platelet integrity. The inhibition of ADP-induced aggregation is used as a major pharmacological principle for the treatment of cardiovascular diseases, for example, after stent implantation in coronary heart disease, associated with an increased risk of bleeding. 16 17 ADP exerts its effects via the purinergic platelet receptors P2Y1, P2Y12, and P2X1. The receptors P2Y1 and P2Y12 are guanine nucleotide-binding protein (G-protein) coupled receptors, whereas P2X1 is an adenosine triphosphate (ATP)-gated, nonselective cation channel. 18 P2Y1 is a G _q_ -coupled receptor, activating platelet phospholipase C, and stimulating calcium release from intracellular stores. 19 P2Y12 inhibits platelet adenylyl cyclase through G _αi_ . 18 Simultaneous activation of both P2Y1 and P2Y12 results in platelet aggregation. 20 Stimulation of the P2X1 receptor alone causes a rapid calcium influx in platelets synergizing P2Y1 effects, but not inducing platelet aggregation. 21

In this study, we analyzed the effects of cold storage on ADP-mediated responsiveness in apheresis-derived PC (APC) compared with room temperature storage, addressing purinergic receptor expression and function, aggregation responses, activation markers, and, in addition, inhibitory signaling pathways.

## Materials and Methods

### Materials

ADP was obtained from Haemochrom Diagnostica GmbH (Essen, Germany), thrombin receptor activating peptide-6 (TRAP-6) from BACHEM (Weil am Rhein, Germany). Mouse monoclonal fluorescein isothiocyanate (FITC)-conjugated antifibrinogen antibody was from STAGO Germany (Düsseldorf, Germany) and mouse monoclonal FITC-conjugated anti-CD62P antibody from Acris antibodies GmbH (Herford, Deutschland). Prostaglandin E1 (PGE1), diethylamine NONOate (DEA/NO), acetylsalicylic acid (ASS), probenecid, pluronic F-127, 4-[2-hydroxyethyl]-1-piperazineethanesulfonic acid (HEPES), apyrase, Ponceau S, and FITC-conjugated goat antirabbit antibody were from Sigma-Aldrich Chemie GmbH (Muenchen, Germany). Rabbit polyclonal anti-P2Y1, anti-P2Y12, and anti-P2X1 antibodies were from Alomone Labs (Jerusalem, Israel). The selective P2Y1 receptor agonist [(1 *R* ,2 *R* ,3 *S* ,4 *R* ,5 *S* )-4-[6-Amino-2-(methylthio)-9 *H* -purin-9-yl]-2,3-dihydroxy, bicycle [3.1.0] hex-1-yl]methyl] diphosphoric acid mono ester trisodium salt (MRS2365), the selective antagonist of P2Y1 (1 *R* *,2 *S* *)-4-[2-Iodo-6-(methylamino)-9 *H* -purin-9-yl]-2-(phosphornooxy) bicyclo-[3.1.0]hexane-1-methanol dihydrogen phosphate ester tetraammonium salt (MRS2500), the agonist of P2X1 receptor α,β-methyleneadenosine 5′-triphosphate trisodium salt (α,β-MeATP), and the potent P2X1 antagonist 4,4 *'* ,4 *''* ,4 *'''* -[carbonylbis (imino-5,1,3-benzenetriyl- *bis* (carbonylimino))] *tetrakis* -1,3-benzenedisul-fonic acid, octasodium salt (NF449) were from R&D Systems GmbH (Wiesbaden-Nordenstadt Germany). Fluo-4AM cell permeant was from Life Technologies GmbH (Darmstadt, Germany). Flow cytometric PLT vasodilator-stimulated phosphoprotein (VASP)/P2Y12 Kit for the measurement of P2Y12 receptor function was from STAGO GmbH (Duesseldorf, Germany). Mouse monoclonal phospho-VASP Ser ^239^ and phospho-VASP Ser ^157^ antibodies were from Nanotools (Teningen, Germany). StarBright Blue 700 conjugated goat antirabbit and antimouse antibodies were from Bio-Rad Laboratories, Inc. (Muenchen, Germany).

### Blood and APC Collection

Venous peripheral blood (PB) samples and apheresis-derived platelet concentrates (APCs) were obtained from informed healthy voluntary donors without any drug intake. Our studies with human platelets and the consent procedure were approved by our local ethics committee of the University of Wuerzburg (approval number: 101/15). The participants provided their written informed consent to participate in this study. The study was performed according to our institutional guidelines and to the Declaration of Helsinki.

PB was collected in polypropylene tubes containing 3.2% citrate buffer (106 mM trisodium citrate, Sarstedt, Nuembrecht, Germany). APC pairs (2.5 × 10 ^11^ platelets in 250 mL of plasma) were collected using Trima Accel devices with version 11.3 software and the Trima Accel LRS Platelet, Plasma Set (Terumo BCT, Lakewood, Colorado, United States). The ratio of inlet blood volume to anticoagulant (ACD-A) was 10:1. After preparation, APCs were stored either at room temperature (room temperature–stored APC, RT-APC) or at 4°C (cold-stored APC [C-APC]) for 6 days according to blood bank conditions on a standard agitator. On days 0 (2–3 hours after finalized apheresis), 2, and 5, samples from APC were taken for analysis under sterile conditions. For aggregation studies, samples were separately drawn on days 1 and 6 due to organizational reasons. Analysis of PB samples on day 0 was started within 1 hour after blood collection.

### Blood Gas Analysis and Platelet Count

Basic characteristics were detected with the blood gas system cobas b 123 POC, software version 4.14 (Roche Diagnostics GmbH, Mannheim, Germany), platelet count with the hematology analyzer KX21N (Sysmex GmbH, Norderstedt, Germany).

### Preparation of Platelet-Rich Plasma and Washed Human Platelets

Platelet-rich plasma (PRP) and washed platelets were prepared as described. 22 Briefly, PRP was obtained by PB centrifugation at 280 g for 5 minutes. EGTA of 3 mM was added to PRP or to samples from APC to prevent platelet activation. Subsequently, samples of PRP and APC were centrifuged at 430 g for 10 minutes. The pelleted platelets were washed once in CGS buffer (120 mM sodium chloride, 12.9 mM trisodium citrate, 30 mM D-glucose, pH 6.5) and resuspended in HEPES buffer (150 mM NaCl, 5 mM KCl, 1 mM MgCl2, 10 mM D-glucose, and 10 mM HEPES, pH 7.4) to a final concentration of 3 × 10 ^8^ platelets/mL.

### Platelet Aggregation

ADP-induced platelet aggregation of 10 µM was measured in PRP or material from stored APC (diluted with plasma to platelet concentration of PRP) using an APACT 4004 aggregometer (LabiTec, Ahrensburg, Germany). Aggregation was measured for 5 minutes under continuous stirring at 1,000 rpm and 37°C. Maximal values were used for statistical calculations.

### Flow Cytometric Analysis

Flow cytometric analysis was performed with PRP or with material from stored APC. For determination of basal and TRAP-6-stimulated fibrinogen binding, 15 µL of PRP or APC samples, diluted with plasma to the platelet concentration in PRP, were stained with 15 µL of FITC-conjugated antifibrinogen antibody or isotype control for 10 minutes at 37°C. After that, the samples were stimulated for 2 minutes at 37°C with buffer (control) or 10 µM TRAP-6.

For determination of basal and TRAP-6-stimulated CD62P expression, 30 µL of PRP or APC samples, diluted with plasma to the platelet concentration in PRP, were stained with 3 µL of FITC-conjugated anti-CD62P antibody or isotype control for 10 minutes at 37°C. After that, the samples were stimulated for 2 minutes at 37°C with buffer (control) or 10 µM TRAP-6.

The reactions were stopped for both fibrinogen- and CD62P-stained samples by fixation for 10 minutes at RT with 1% formaldehyde (final concentration). After that, they were diluted with 300 µL of PBS/BSA/Glc and analyzed by flow cytometry.

For flow cytometric detection of purinergic receptor surface expression, 30 µL of PRP or APC samples, diluted with plasma to the platelet concentration in PRP, were stained with 3 µL of anti-P2Y1, anti-P2Y12, or anti-P2X1 antibodies or isotype control for 10 minutes at 37°C. After that, the samples were stimulated for 2 minutes at 37°C with buffer (control) or 10 µM TRAP-6. Samples were stopped with 1% formaldehyde (final concentration), fixed for 10 minutes at RT, and then centrifuged for 1 minute at 14,000 g. The pellet was resuspended in 100 µL of PBS/BSA/Glc (Dulbecco's PBS (Ca ^2+^ , Mg ^2+^ free), 5.5 mM D-glucose, 0.5% BSA) and stained at RT in the dark for 30 minutes with 1 µL of FITC-conjugated goat antimouse antibody. After that samples were diluted with 300 µL of PBS/BSA/Glc and analyzed by flow cytometry.

Flow cytometric analysis was performed on a FACS Calibur flow cytometer from Becton Dickinson (Franklin Lakes, New Jersey, United States) using CELLQuest software, version 6.0.

The platelet population was identified by its forward and side scatter distribution and 10,000 events were analyzed for mean fluorescence.

### Platelet Preparation for the Measurement of P2Y1 Activity

PGE1 of 500 nM was added to PRP (as described for the preparation of washed platelets) or to material from stored APC and then centrifuged at 430 g for 10 minutes. The pellet was washed with 5 mL of modified Tyrode's buffer (10 mM HEPES, 150 mM NaCl, 3 mM KCl, 1 mM MgCl _2_ , 5 mM glucose and 0.1% BSA, pH 6.5) containing 500 nM PGE1. Platelets were resuspended in modified Tyrode's buffer without PGE1 and platelet concentration was adjusted to 0.6 × 10 ^8^ platelets/mL. 23

### Platelet Preparation for the Measurement of P2X1 Activity

ASS of 1 mM and 0.3 U/mL apyrase were added to PRP (as described for the preparation of washed platelets) or to material from stored APC and then centrifuged at 430 g for 10 minutes. The pellet was washed with 5 mL of modified Tyrode's buffer containing 1 mM ASS and 0.3 U/mL apyrase. Platelets were resuspended in modified Tyrode's buffer containing 0.3 U/mL apyrase and platelet concentration was adjusted to 0.6 × 10 ^8^ platelets/mL. 23

### Measurement of P2Y1 and P2X1 Activity

The activity of platelet purinergic P2Y1 and P2X1 receptors was measured by calcium flux-induced fluorescence in Fluo-4AM-loaded platelets after selective stimulation. 23 Briefly, in each well of a 96-well black plate, 100 µL of washed platelets were mixed with an equal volume of Hank's buffered saline solution (HBSS) containing 10 mM HEPES, 0.1% BSA, 2.5 mM probenecid, 1 mM EGTA, 0.01% pluronic acid, and 2 µM Fluo-4AM at pH 7.4.

For the measurement of P2X1 activity, EGTA was substituted by 2.5 mM calcium and apyrase was added to the final concentration of 0.3 U/mL. The plate was incubated for 20 minutes at RT in the dark, followed by 20 minutes of incubation at 37°C. During the last 10 minutes of incubation, 2 µL of 100 µM MRS2500, a P2Y1 antagonist, or 2 µL of 100 µM NF 449, a P2X1 antagonist, were added in negative controls. After measurement of the basal fluorescence (Ex 488, Em 538; 20 measurements at 1 second), platelets were stimulated with 2 µL of 100 µM MRS2365, a P2Y1 agonist, or 2 µL of 100 µM α, β-MeATP, a P2X1 agonist. After stimulation, fluorescence signals were measured every second for the next 3 minutes using Fluoroscan Ascent Microplate Fluorometer from Fisher Scientific GmbH (Schwerte, Germany).

### Measurement of P2Y12 Activity

The activity of platelet P2Y12 receptor was measured by the flow cytometric PLT VASP/P2Y12 Kit. Briefly, aliquots of PB or APC, diluted with plasma to 3 × 10 ^8^ platelets/mL were stimulated with PGE1 alone or with a combination of PGE1 and ADP at RT. After stimulation, samples were fixed and stained as described in the manufacturer's instructions, followed by flow cytometric measurement of fluorescence. Platelet reactivity index (PRI) was calculated using corrected mean fluorescence intensities (MFIc) as PRI = [MFIc (PGE1) – MFIc (PGE1 + ADP)] / [MFIc (PGE1)] × 100%.

### Western Blot Analysis

VASP phosphorylation in washed platelets was determined by Western blot analysis. For this purpose, 100 µL of washed platelet suspension was supplemented with 1 mM CaCl _2_ , followed by stimulation with buffer, 5 nM DEA/NO, or 5 nM PGE1 for 2 minutes at 37°C. The cell lysates were loaded onto the gel, separated by sodium dodecyl sulfate polyacrylamide gel electrophoresis (SDS-PAGE) and then transferred onto nitrocellulose membranes. The membranes were incubated with mouse monoclonal phospho-VASP Ser ^239^ (Clone 16C2) and phospho-VASP Ser ^157^ (Clone 5C6) antibodies overnight at 4°C. For visualization of the signal, goat antimouse immunoglobulin (Ig)-G conjugated with StarBright Blue 700 was used as secondary antibody, followed by detection with Chemidoc MP imaging system (Bio-Rad Laboratories, Inc., Hercules, California, United States) and analysis with the corresponding Image Laboratory Software Version 6.0.

### cAMP and cGMP Measurements

Cyclic adenosine monophosphate (cAMP) and cyclic guanosine monophosphate (cGMP) in washed platelets were detected by cAMP (enzyme-linked immunoassay) ELISA Kit and GMP ELISA Kit, respectively, following the manufacturer's instructions (Cayman Chemical, Hamburg, Germany).

### Statistical Analysis

Descriptive data were calculated with the MedCalc statistic program (MedCalc Software bvba, Mariakerke, Belgium) and GraphPad PRISM 7 (GraphPad Software, San Diego, California, United States). Data distribution analysis was performed with Shapiro–Wilk test. Differences of variances between groups were analyzed by one-way analysis of variance (ANOVA) followed by post hoc Tukey–Kramer test. A *p* < 0.05 was considered statistically significant, *p* < 0.1 as tendency.

## Results

### ADP-Induced Aggregation Preservation in Cold-Stored Platelets

**Fig. 1:**
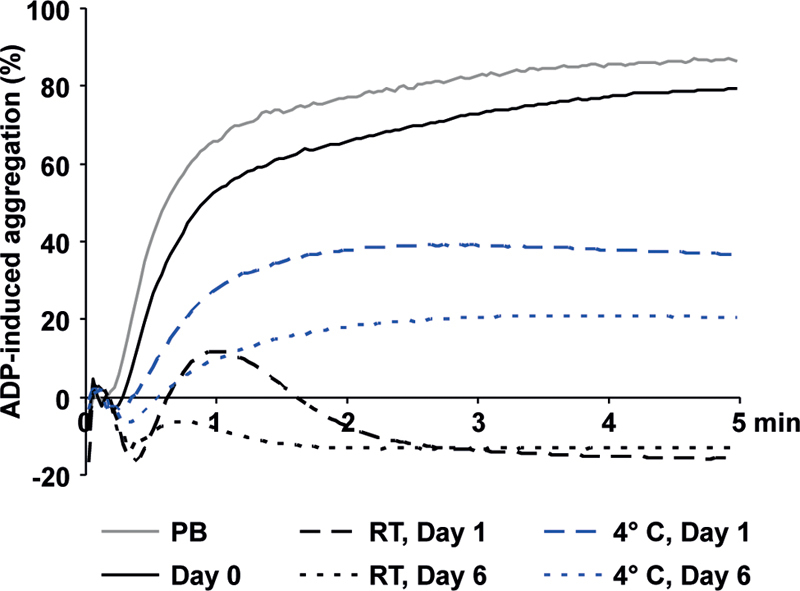
ADP-induced aggregation under RT and cold storage. PRP of fresh PB samples, of RT-APC and of C-APC was used to measure light transmission aggregation after stimulation with 10 µM ADP. Mean aggregation curves are shown. *n* = 6. ADP, adenosine diphosphate; C-APC, cold-stored apheresis-derived platelet concentrates; RT, room temperature.

In freshly prepared APCs, 10 µM ADP-induced light transmission aggregation showed values of approximately 80%, comparable to PRP of PB samples ( Fig. 1 ). The aggregation responses in RT-APC were dramatically decreased on day 1 and completely declined until day 6. In contrast, aggregation was partially maintained in C-APC with 36.6 ± 11.9% on day 1 and 20.4 ± 12.1% on day 6.

### Preactivation Marker Increase under Cold Storage

**Fig. 2:**
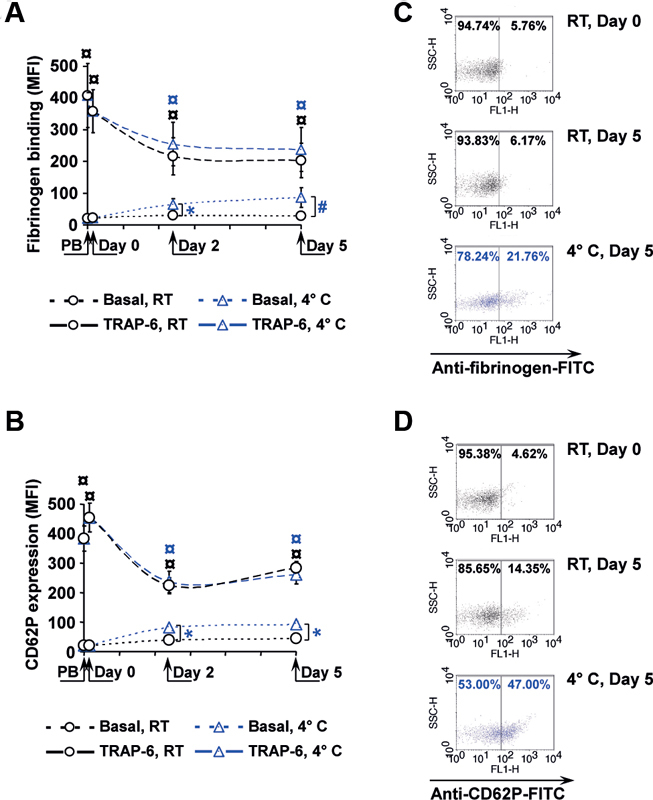
CD62P expression and fibrinogen binding. Basal or 10 µM TRAP-6-induced CD62P expression ( **A** ) and fibrinogen binding ( **B** ) were measured by flow cytometry at different time points as indicated. Representative scatter diagrams illustrate the distribution of platelets detected by FITC-conjugated antifibrinogen antibodies ( **C** ) or anti-CD62P antibodies ( **D** ). *n* = 4; mean ± SEM; ^#^ *p* < 0.1 or * *p* < 0.05, compared with RT-APC at the same time points; ^¤^ *p* < 0.05, compared with basal values (for RT-APC in black, for C-APC in blue). C-APC, cold-stored apheresis-derived platelet concentrates; MFI, mean fluorescence intensities; RT-APC, room temperature APC; SEM, standard error of mean; TRAP-6, thrombin receptor activating peptide.

The reactivity and the preactivation status of stored platelets were analyzed by basal and induced fibrinogen binding and CD62 expression ( Fig. 2 ). Upon stimulation with 10 µM TRAP-6, fibrinogen binding in PB samples and in APCs increased from 21.7 ± 1.0 to 408.7 ± 100.9 MFI, and from 23.0 ± 1.5 to 359.1 ± 67.7 MFI, respectively ( Fig. 2A ). On days 2 and 5, the basal values remained unchanged in RT-APC, but increased to 65.4 ± 17.3 and 86.8 ± 31.0 MFI in C-APC. TRAP-6-stimulated values decreased in the course of storage to 216.5 ± 58.9 MFI on day 2 and to 203.4 ± 54.1 MFI on day 5 for RT-APC, and comparably to 254.7 ± 67.3 MFI and 238.4 ± 69.0 MFI in C-APC. Basal CD62P expression rose during storage of C-APC from 20.6 ± 0.8 MFI to 93.1 ± 8.9 MFI until day 5, and to 44.9 ± 5.9 MFI in RT-APC on day 5 ( Fig. 2B ). The increment of TRAP-6-stimulated CD62P expression was decreasing until day 5 under both storage conditions, with 454.5.7 ± 49.0 MFI on day 0 and 283.6 ± 22.8 MFI on day 5 for RT-APC, and 260.6 ± 30.7 MFI on day 5 for C-APC. Representative flow cytometry dot plot diagrams of sideward light scatter (SSC) versus FITC fluorescence (FL1) illustrate the shift of unstimulated platelets toward elevated fibrinogen binding and CD62P expression in C-APC in contrast to RT-APC ( Fig. 2C and D ).

### Basic Characteristics of C-APC and RT-APC

C-APC developed a slight reduction of platelet concentration and a mild increase of potassium levels during storage in comparison to RT-APC ( Table 1 ). The pH levels measured at 22°C showed a rising tendency, more pronounced in RT-APC. Glucose consumption and lactate generation was stronger in RT-APC, whereas annexin V binding remained unchanged during storage at both temperatures.

**Table 1:** Basic characteristics of C-APC and RT-APC

Parameter	Unit	APC (day 0)	RT-APC (day 2)	RT-APC (day 5)	C-APC (day 2)	C-APC (day 5)
Platelets	×10 ^3^ /µL	1,148 ± 49	1,145 ± 68	1,132 ± 66	1,006 ± 126 a, c	952 ± 145 a, c
Potassium	mmol/L	3.18 ± 0.08	3.24 ± 0.04	3.26 ± 0.04 a	3.45 ± 0.06 b, d	3.52 ± 0.03 b, d
Annexin V	MFI	17.3 ± 2.4	18.2 ± 1.5	21.5 ± 1.4	18.2 ± 2.4	18.1 ± 1.0
pH (22°C)	–	7.37 ± 0.03	7.60 ± 0.02 b	7.46 ± 0.03	7.47 ± 0.02 a, d	7.45 ± 0.02 a
Glucose	mg/dL	399 ± 36	369 ± 39 b	329 ± 44 b	388 ± 37 b, d	372 ± 41 b, d
Lactate	mmol/L	1.90 ± 0.37	4.63 ± 0.32 b	9.03 ± 0.47 b	3.43 ± 0.29 b, d	5,90 ± 0.21 b, d

Abbreviations: C-APC, cold-stored apheresis-derived platelet concentrates; MFI: mean fluorescence intensity (in arbitrary units);RT-APC, room temperature APC.

a *p* < 0.1, compared with day 0.

b *p* < 0.05, compared with day 0.

c *p* < 0.1, compared with the corresponding day of storage.

d *p* < 0.05, compared with the corresponding day of storage; mean ± standard error of mean; *n* = 3.

### Purinergic Receptor Expression Increases, but the Function of P2Y1 and P2X1 Decreases during Cold Storage

**Fig. 3:**
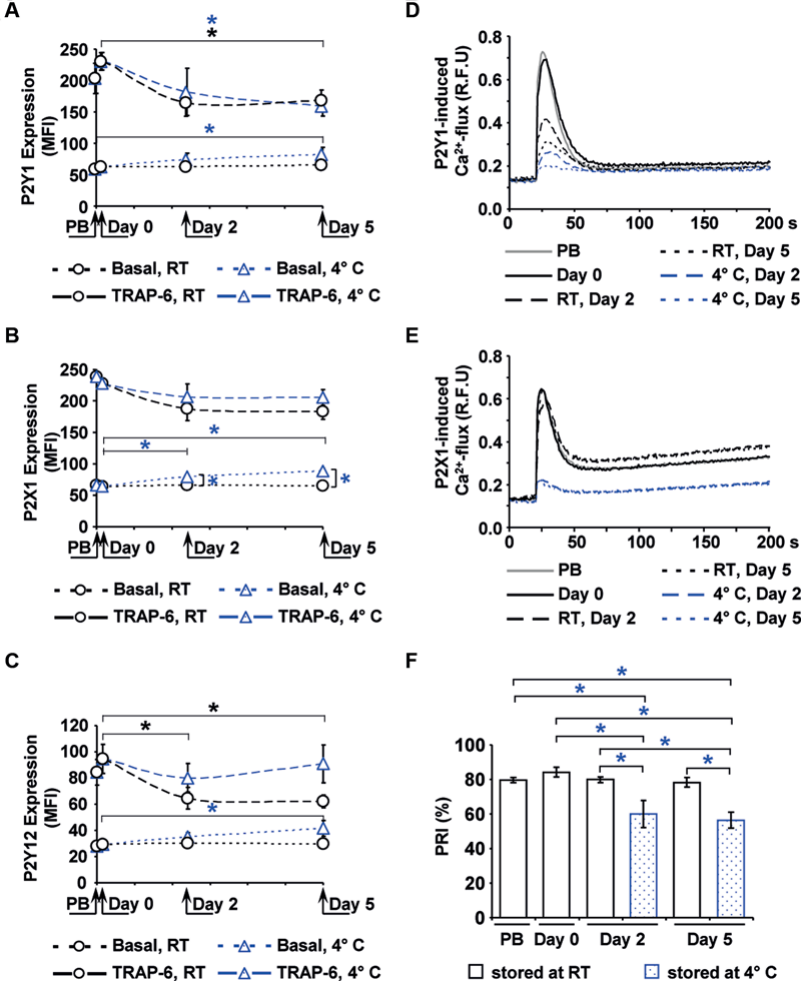
Expression and function of purinergic receptors. The mean fluorescence intensities (MFI) of basal or 10 μM TRAP-6-stimulated receptor expression, detected by flow cytometry, are shown for P2Y1 ( **A** ), P2Y12 ( **B** ) and P2X1 ( **C** ). The function of the P2Y1 ( **D** ) and of the P2X1 ( **E** ) receptor was measured by calcium-induced fluorescence after selective stimulation, shown as mean values in relative fluorescence units (RFU). The function of the P2Y12 receptor ( **F** ) is expressed as platelet reactivity index (PRI) determined by the flow cytometric VASP assay. Mean ± SEM; *n* = 6; * *p* < 0.05 (for RT-APC in black, for C-APC in blue). C-APC, cold-stored apheresis-derived platelet concentrates; PB, peripheral blood; RT, room temperature; SEM, standard error of mean; TRAP-6, thrombin receptor activating peptide; VASP, vasodilator-stimulated phosphoprotein.

Since ADP responsiveness of platelets is mediated via purinergic receptors, the expression and function of P2Y1, P2X1 and P2Y12 receptors were analyzed ( Fig. 3 ). In fresh APC, the basal expression remained unchanged for all three receptor types compared with PB ( Fig. 3 A–C ). In contrast to RT-APC with stable values throughout storage, the basal expression slightly increased in C-APC until day 5, by 31% for P2Y1, by 37% for P2X1, and by 43% for P2Y12. Initially, TRAP-6 stimulated purinergic receptor expression by approximately four folds. In the course of storage, induced P2Y1 expression decreased by 20% in both APC types. P2X1 expression was comparably maintained, whereas P2Y12 expression dropped in RT-APC by 25% until day 5 in contrast to stable levels in C-APC.

P2Y1 function, measured by calcium-induced fluorescence after selective stimulation, showed a progressive decline in C-APC from 0.69 ± 0.10 to 0.26 ± 0.02 relative fluorescence units (RFU) on day 2 and to 0.20 ± 0.01 RFU on day 5. In RT-APC, this effect was less emphasized with 0.42 ± 0.05 RFU on day 2 and 0.31 ± 0.02 RFU on day 5 ( Fig. 3D ). P2X1-related calcium-induced fluorescence remained almost unaffected during storage in RT-APC, but dropped in C-APC from 0.65 ± 0.08 to 0.22 ± 0.01 RFU on day 2 and 0.22 ± 0.02 RFU on day 5 ( Fig. 3E ). The PRI levels indicating P2Y12 activity were stable with approximately 80% throughout storage in RT-APC, comparable to PB samples. However, PRI levels in C-APC dropped to 60.1 ± 7.7% on day 2 and to 56.4 ± 4.6% on day 5 ( Fig. 3F ).

### P2Y12 Function is Maintained in Cold-Stored Platelets

**Fig. 4:**
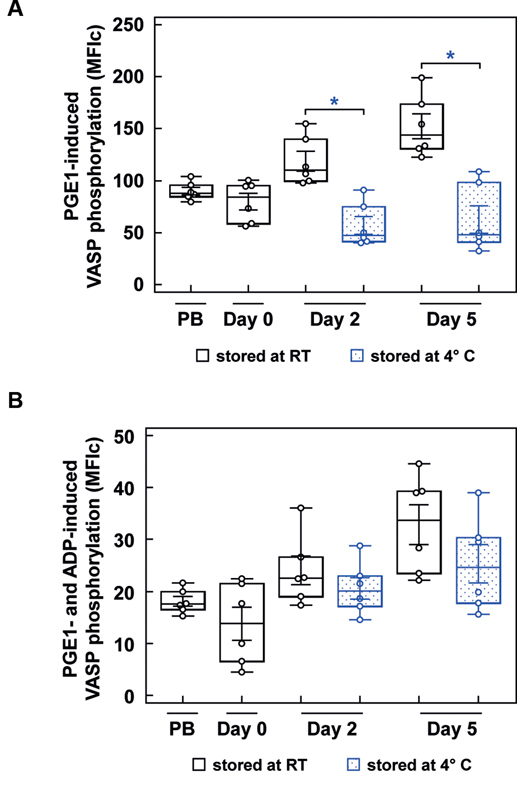
PRI-related VASP phosphorylation measured by flow cytometry. The box-and-whisker plots show the distribution of corrected mean fluorescence intensities after stimulation with PGE1 ( **A** ) and after stimulation with PGE1 + ADP ( **B** ) of PB samples, RT-APC and C-APC (blue color) as indicated; Analysis was performed by flow cytometry. Results are presented as mean ± SEM; *n* = 6; * *p* < 0.05. ADP, adenosine diphosphate; C-APC, cold-stored apheresis-derived platelet concentrates; MFI, mean fluorescence intensities; PB, peripheral blood; PGE1, prostaglandin E1; PRI, platelet reactivity index; RT, room temperature; SEM, standard error of mean; VASP, vasodilator-stimulated phosphoprotein.

The PRI is a calculated parameter using PGE1-induced VASP phosphorylation levels without or with additional ADP stimulation ( Fig. 4 ). In contrast to RT-APC, PGE1-induced VASP phosphorylation was significantly decreased in C-APC reaching 57.0 ± 9.3 corrected mean fluorescence intensity (MFIc) on day 2 and 62.7 ± 14.4 MFIc on day 5 of storage ( Fig. 4A ). The additional stimulation with ADP led to an excessive decrease of VASP phosphorylation to levels as measured in RT-APC ( Fig. 4B ), indicating an unaffected functional activity of the P2Y12 receptor.

### VASP Phosphorylation is Less Inducible in Cold-Stored Platelets

**Fig. 5:**
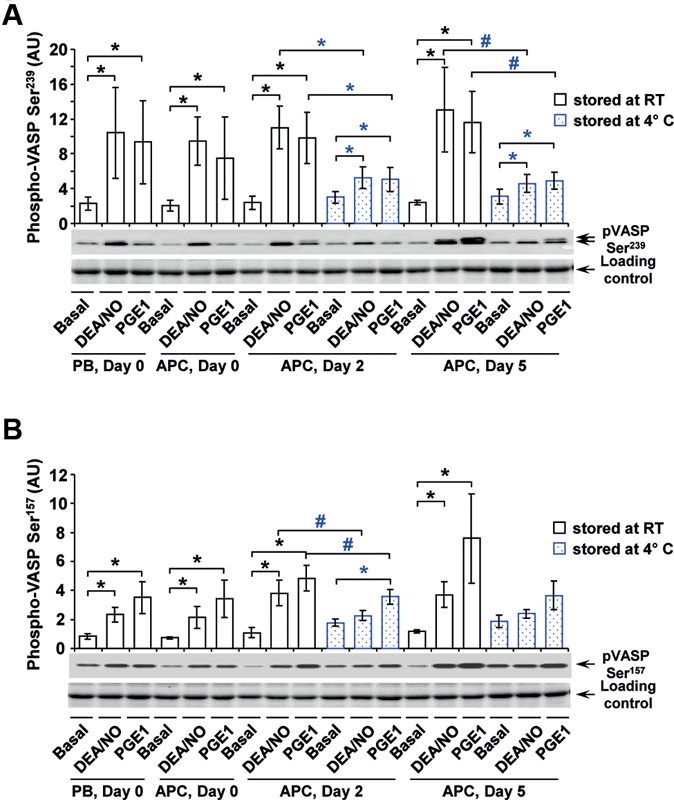
Western blot analysis of basal and induced VASP phosphorylation. Quiescent washed platelets from fresh PB samples and from stored RT-APC or C-APC, without or after stimulation with DEA/NO or PGE1 as indicated, were lysed with Laemmli's buffer and analyzed on Western blot for VASP Ser ^239^ ( **A** ) and VASP Ser ^157^ ( **B** ) phosphorylation. After scanning bands were quantified by the Image Lab program. Results are presented in arbitrary units (AU) as mean ± SEM; *n* = 5; * *p* < 0.05, ^#^ *p* < 0.1 (compared as indicated; blue: RT-APC to C- APC). C-APC, cold-stored apheresis-derived platelet concentrates; DEA/NO, diethylamine NONOate; PB, peripheral blood; PGE1, prostaglandin E1; RT, room temperature; SEM, standard error of mean; VASP, vasodilator-stimulated phosphoprotein.

The inhibitory pathways of platelets were investigated by Western blot analysis of VASP phosphorylation at Ser ^239^ and Ser ^157^ using the nitric oxide (NO) donor diethylamine NONOate (DEA/NO) and prostaglandin E1 (PGE1; Fig. 5 ). In RT-APC, 5 nM DEA/NO and PGE1 similarly provoked a 4–5-fold increase of phosphorylation at Ser ^239^ with increasing tendency during storage ( Fig. 5A ). In C-APC, the increments at Ser ^239^ were smaller, with a 1.8-fold elevation on day 2 and a 1.6-fold elevation on day 5 for DEA/NO stimulation, and 1.5-fold and 1.6-fold PGE1 stimulation, respectively. At Ser ^157^ , phosphorylation levels were similarly stimulated in RT-APC, up to three folds under DEA/NO or four folds under PGE1 compared with basal values ( Fig. 5B ). In C-APC, induced phosphorylation was less emphasized, with no significant increase on both investigated days for DEA/NO stimulation and a significant, but only two-fold shift on day 2 for PGE1.

### Stimulation of Cyclic Nucleotide Levels is Dampened under Cold Storage

**Fig. 6:**
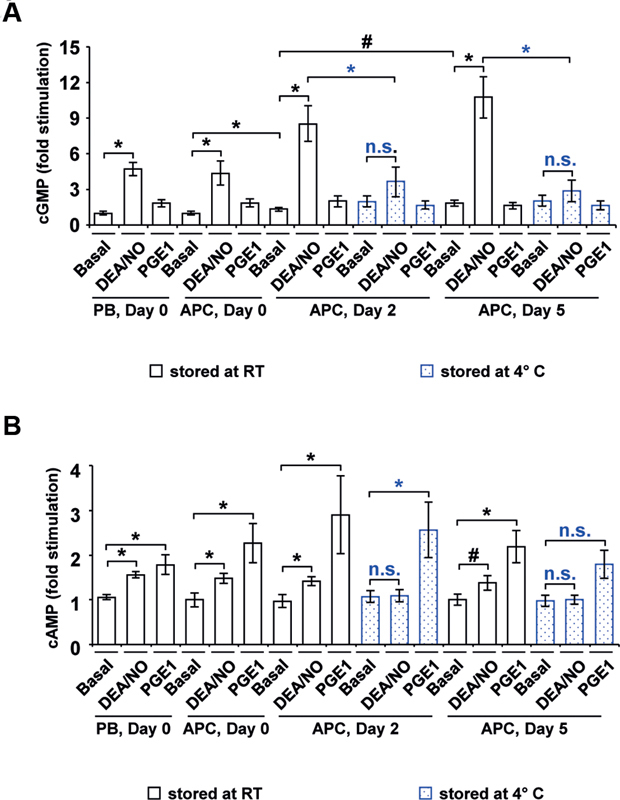
Cyclic nucleotide levels. Cyclic nucleotide concentrations were measured in lysed washed platelets from fresh PB samples and from stored RT-APC or C-APC. After extraction with diethyl ether the contents of cGMP ( **A** ) and cAMP ( **B** ) were determined with immunoassays. Results are presented as mean (fold stimulation) ± SEM; *n* = 4; * *p* < 0.05, ^#^ *p* < 0.1 (compared as indicated; blue: RT-APC to C-APC; n.s.: not significant). APC, apheresis-derived platelet concentrates; cAMP, cyclic adenosine monophosphate; C-APC, cold-stored apheresis-derived platelet concentrates; cGMP, cyclic guanosine monophosphate; DEA/NO, diethylamine NONOate; PB, peripheral blood; PGE1, prostaglandin E1; RT, room temperature; SEM, standard error of mean.

The cyclic nucleotide levels (cAMP and cGMP) were additionally measured to complement data from Western blot analysis ( Fig. 6 ). In RT-APC, DEA/NO increased cGMP concentrations more than four folds on day 0, comparable to PB samples, 8.5 folds on day 2 and 11 folds on day 5 ( Fig. 6A ). In contrast, DEA/NO was not able to relevantly enhance cGMP levels in C-APC. PGE1 did not affect cGMP values, but led to an elevation of cAMP levels up to two folds on days 2 and 5 in RT-APC ( Fig. 6B ). The increment of cAMP concentrations continuously decreased during storage of C-APC. For the stimulation with DEA/NO, a weak induction of cAMP levels by 50% was observed in RT-APC at all time points of storage, but not in C-APC.

### The Inhibitory Effect of DEA/NO and PGE1 is Reduced in Cold-Stored Platelets

**Fig. 7:**
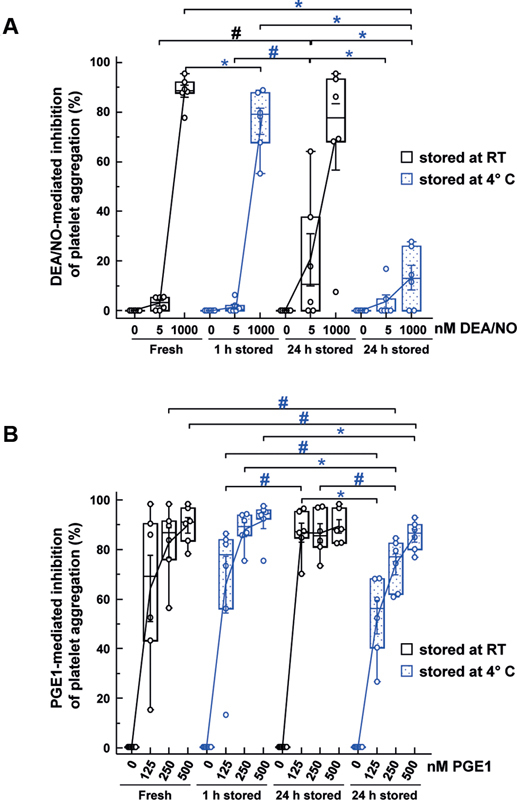
Inhibitory effects of DEA/NO and PGE1 on ADP-induced aggregation in RT or cold-stored PB samples. Light transmission aggregation was performed with PRP of stored PB samples as indicated. The results are shown as mean % ± SEM and represent the relative inhibition of 10 µM ADP-induced aggregation in relation to the corresponding PB samples without DEA/NO or PGE1 incubation (as 0% inhibition); *n* = 6; * *p* < 0.05, ^#^ *p* < 0.1 (compared as indicated; blue: RT-APC to C-APC). ADP, adenosine diphosphate; C-APC, cold-stored apheresis-derived platelet concentrates; DEA/NO, diethylamine NONOate; PB, peripheral blood; PGE1, prostaglandin E1; RT, room temperature; SEM, standard error of mean.

DEA/NO- or PGE1-mediated inhibition of ADP-induced aggregation is attenuated in platelets from PB samples stored as citrated whole blood at 4°C for 24 hours ( Fig. 7 ). Compared with fresh PB samples with unaffected aggregation under 5 nM DEA/NO (2.9% inhibition) and almost complete inhibition (more than 88.5% inhibition) under 1,000 nM, DEA/NO resulted in 20.5 ± 11.6% inhibition at 5 nM and 70.0 ± 14.6% inhibition at 1,000 nM after 24 hours of RT-storage ( Fig. 7A ). Under cold storage for 24 hours, the inhibition of aggregation was dramatically reduced to 3.6 ± 3.0% at 5 nM DEA/NO and 13.3 ± 5.4% at 1,000 nM DEA/NO compared with the extent of inhibition in freshly drawn, 1 hour cold or 24 hours RT-stored samples. The onset of tampered inhibition is observable after 1 hour of cold storage with significantly weaker inhibition of aggregation by 76.3 ± 5.7% at 1,000 nM DEA/NO in comparison to inhibition of ADP-induced aggregation in freshly prepared PB samples.

PGE1 of 125 nM led to 64.0 ± 14.6% inhibition of ADP-induced aggregation, whereas 250 and 500 nM PGE1 almost completely blocked it in fresh PB ( Fig. 7B ). In samples stored for 24 hours at RT, all investigated PGE1 concentrations prevented ADP-induced platelet aggregation, whereas cold storage was associated with lower inhibitory effects after 1 hour with 65.6 ± 12.6% at 125 nM and with 87.6 ± 3.1% at 250 nM PGE1, and after 24 hours even more pronounced with 52.3 ± 7.4% or 73.7 ± 4.6%, respectively. At the high 500 nM PGE1 concentration, there was no reduction of inhibition after 1 hour and it was only slightly tampered with 85.1 ± 2.7% after 24 hours at cold temperature compared with fresh PB samples with 89.5 ± 3.5%.

## Discussion

The minimization of storage lesions by optimization of storage conditions is an important issue of manufacturing platelet concentrates. 24 25 26 In this context, it was the intention to analyze the effects of cold temperature on ADP-dependent responsiveness, a system playing an important role for physiological platelet integrity and used as pharmacological target to suppress thrombus formation. 16 17

The study confirms that ADP-induced platelet aggregation is better maintained at 4°C in comparison to RT. 25 27 28 The molecular and biochemical investigations have revealed that the mechanisms for increased ADP responses are related to cold-induced attenuation of inhibitory signaling rather than to differences in expression or function of purinergic receptors. In general, cold storage leads to preactivation of platelets 9 11 as illustrated by elevated basal levels of fibrinogen binding and CD62P expression. Hoffmeister et al could show that rearrangements of the GPIb surface configuration are initiated by cold temperature, leading to its clustering. 29 Therefore, it would be of interest, if purinergic receptors experience a similar variation of their assembly on the platelet surface. An essential influence of refrigeration on purinergic receptor expression, however, was not observable, although the surface content of these receptors increase upon agonist-induced platelet activation. 30

Surprisingly, the functional activity of P2Y1 and P2X1 measured as calcium-induced fluorescence was even less maintained at cold storage. Earlier studies have also shown that refrigeration disturbs the internal calcium regulation of platelets. 31 Instead, the P2Y12 receptor appears to be more robust to storage lesion at RT 32 and similarly at 4°C. The coupling of the receptor to intraplatelet signaling pathways other than P2Y1 and P2X1, without association to calcium release, may play a role for that phenomenon. In the context of PRI determination, it is required to closely study the underlying shifts of VASP phosphorylation. 33 34 Thereby, it was evident that stimulation of PGE1-induced VASP phosphorylation in C-APC is decreased, whereas ADP-induced inhibition of VASP phosphorylation remains intact, indicating maintained P2Y12 receptor function.

PGE1 stimulates one of the two main inhibitory pathways in platelets via the platelet IP (prostacyclin) receptor, coupled to G _s_ -activating protein of membrane-associated adenylyl cyclase (AC). AC activation leads to the increase of intracellular cAMP concentration followed by enhanced cAMP-mediated VASP phosphorylation. 35 36

The second inhibitory pathway in platelets is activated by NO produced by various NO synthase-containing cells or released by chemical compounds like DEA/NO. 35 36 NO permeates through the platelet membrane and directly activates soluble guanylyl cyclase (sGC) in the cytosol. This results in the stimulation of cGMP production and, consecutively, in enhanced cGMP-mediated VASP phosphorylation. 35 36 In C-APC, VASP phosphorylation was less inducible at both Ser ^239^ and at Ser ^157^ after PGE1 and DEA/NO stimulation. Accordingly, intracellular levels of cAMP and cGMP, stimulated with PGE1 or DEA/NO, were reduced in C-APC compared with RT-APC.

Despite different molecular mechanisms and different substrates like ATP and GTP, both inhibitory pathways in platelets are affected by cold storage. However, the key enzymes AC and sGC possess the same pyrophosphatelyase (cyclizing) activity, (ATP pyrophosphatelyase [cyclizing], EC 4.6.1.1) and (GTP pyrophosphatelyase [cyclizing], EC 4.6.1.2), 37 consistent with cognate thermosensitive characteristics. In former studies, it was demonstrated that the increase of temperature for enzyme incubation from low to higher than 25°C led to an abrupt decrease of energy of AC activation. 38 The enzyme stability and the rate of AC activation from *Neurospora grassa* and turkey erythrocytes were also temperature and membrane lipid composition (fluidity) dependent. 39 40 Similar to AC, GC enzyme activities from various species (human embryonic kidney cells [HEK-293T], *Caenorhabditis elegans* , and monkey fibroblast-like cell line Cos-7) were sensitive to temperature variations with activity peaks between 15 and 30°C. 41 42 43 Platelet storage at 4°C for more than 24 hours may have an impact not only on enzyme kinetic, but also induce a long-term change of membrane and surrounding proteins fluidity. These effects may result in irreversible dysfunction of pyrophosphatelyase (cyclizing) activity of AC and GC, reduced cAMP and cGMP synthesis, and as a consequence, in impaired VASP phosphorylation and platelet inhibition.

## Limitations

As a limitation, it should be mentioned that the results refer to an in vitro study with platelets separated by apheresis and stored in autologous plasma. However, platelet concentrates can be manufactured with variable methods, for example, as pooled concentrates, with the use of additive solutions or treated by pathogen inactivation procedures. 44 45 The impact of cold storage on ADP-dependent platelet function in such product modifications should be subject of additional studies. In this study, the standard ACD-A solution was used as the common anticoagulant in transfusion medicine, albeit the citrate-induced reduction of ionized calcium potentially interferes with platelet function. Thrombin inhibitors-like hirudin may be alternatives, but there is evidence that thrombin inhibitors also affect inhibitory pathways, thereby enhancing VASP phosphorylation and dampening platelet reactivity. 34 Interestingly, BAPA (benzylsulfonyl-D-Arg-Pro-4-amidinobenzylamide) represents a dual inhibitor of factor Xa and thrombin, was able to maintain platelet aggregation and function in stored blood samples at RT better than citrate, indicating a significant role of the anticoagulant in platelet preservation. 46 47

## Conclusion

In conclusion, cold-stored platelets possess a higher sensitivity to ADP despite impairment of P2Y1 and partially of P2X1 function. Attenuated inhibitory signaling with early onset during cooling is a major factor promoting activation and ADP-induced aggregation of platelets compared with RT-APC. Future studies should address time-dependent effects of refrigeration on ADP-mediated platelet integrity and the reversibility of cold induced storage lesions to further improve manufacturing processes of platelet concentrates and to facilitate the design of clinical trials with cold-stored platelet products.
